# Can hypothesis‐driven research survive the sequence‐data deluge?

**DOI:** 10.1111/1751-7915.13377

**Published:** 2019-03-19

**Authors:** Mark Sagoff

**Affiliations:** ^1^ Institute for Philosophy and Public Policy George Mason University Fairfax VA USA

*This satirical Opinion questions funding trends that perpetuate massive infrastructures the value of which to science has become increasingly less obvious over time. These funding trends are especially dubious in the case of Genome Centers, which (perhaps unintentionally) discourage hypothesis‐driven research in favour of mindless data generation and speculative hand‐waving about its relevance*.

The scientific world was set agog when a team of biologists at the University of Berserkerstan announced that they had produced, by using CRISPR technology, a half‐man half‐horse. Xiangpo Zucchini, who headed the research team, conceded that the experiment had not gone entirely as intended. ‘We combined a horse embryo with a human embryo’, Zucchini said, ‘but the result, when brought to term by a mare, was mixed‐up. It had the head and torso of a horse and the hindquarters of a human being’.

Zucchini said that nevertheless gene‐drive technology could be added that will breed into the human population more horse sense than it now has.

Etta Whatney, Ph. D., S.P.Q.R., who advises the National Human Genome Research Institute (NHGRI) on ethical issues, said further research was needed to determine whether the Zucchini *et al*. centaur is ‘less human’ than a conventional centaur, which has equine hindquarters and a human front.

The crucial question, Whatney said in a telephone interview, is to determine whether the centaur produced in the Zucchini Lab qualifies as a model organism or as a human subject.

NHGRI awarded $1.5 million to ethicists at Case Western Reserve University ‘to enhance guidelines for ethical human‐animal chimera research’.^1^ Whatney added that Zucchini *et al*., by putting the human posterior aft, created an animal that may produce less methane than a conventional centaur. ‘All project proposals will be required to state their impact on climate change’.

Hal Garmisch, Ph. D., L.M.N.O.P., a colleague of Dr. Whatney, joined the call while travelling to a predatory conference in Kuala Lumpur. ‘Many of the most important medical discoveries result from accidents, for example, when a lab assistant leaves the window open and a mold wafts in and kills bacteria in a petri dish’. Garmisch agreed that the new design of the half‐horse half‐man, although a surprise, could be beneficial. ‘Surprise is often a better teacher than science in medicine’.

White Papers are now in preparation on the feasibility of creating a Minotaur (part human and part bull); an onocentaur (part human, part donkey), a harpy (part woman, part bird); a satyr (part human, part goat); and a mermaid. NHGRI anticipates funding the pushmi‐pullyu (pronounced ‘push‐me—pull‐you’), a cross between a unicorn and a gazelle with two heads (one of each) at opposite ends of its body. This genome could be used to model schizophrenia, capgras syndrome and other maladies.

Animals whose genomes have been sequenced point to the pushmi‐pullyu as a promising model organism for psychiatric research. Dr. John Dolittle, Puddleby‐on‐the‐Marsh, is at the right.

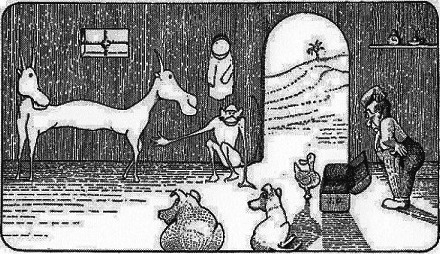

According to its website, NHGRI was created in 1989 to manage the role the National Institutes of Health (NIH) would play in the Human Genome Project (HGP).^2^ The HGP, which began in 1990 at a cost of $5 billion (in inflation‐adjusted dollars), was expected to take 15 years but was finished sooner and under‐budget in part because microbiologist Craig Venter sequenced parts of his own genome for lunch money and carfare.^3^ The HGP was declared complete in April 2003.

By sequencing parts of his own genome, Venter had circumvented the careful protocols for donor selection, anonymization, de‐identification and privacy which regulate publicly funded research. He had not properly secured his informed consent as required under the Common Rule which protects human subjects of research; he had violated his own privacy. Since his project did not receive federal funds, however, he could not be prosecuted for this offense.

Starting in 2011, ethicists and others worked on a new Common Rule to prevent this sort of abuse.^4^ To ensure the privacy of genomic data against all contingencies, however abstruse and far‐fetched, the proposed new Rule would apply to nearly all genomic research, including projects that were not federally funded, making ethics often more challenging than science.^5^

The completion of the HGP early and under‐budget left NHGRI with another grand challenge: an *embarras de richesse*. The last thing a federal agency wants to do is close out a fiscal year with a surplus because it may be dinged for the difference the following year.

‘There remains a compelling need to sequence the genomes of many more organisms’, NHGRI announced at a news release dated 7 November 2003 and budgeted $459 million to establish and support through 2006 the Large‐Scale Sequencing Research Network.^6^ This moved the goalposts and gave the usual PIs another first‐and‐ten.

In November 2003, Hamish Farfel, Ph. D., A.C.G.T., who was interviewed for this article, operated a food truck on the NIH campus after his postdoc expired. He worried that his best‐selling ‘all‐beef’ bratwurst might be sequenced and exposed. He chatted up his customers. Why not sequence ‘man's best friend’? According to Farfel, the lunchtime crowd loved the idea. Dogs are very popular. NHGRI got about $30 million out the door to sequence the genome of a boxer named ‘Tasha’.^7^ Venter had already sequenced his pet poodle Shadow, but only for a small fraction of the cost.^8^

A dog is prepared for sequencing at the NHGRI Dog Genome Project.

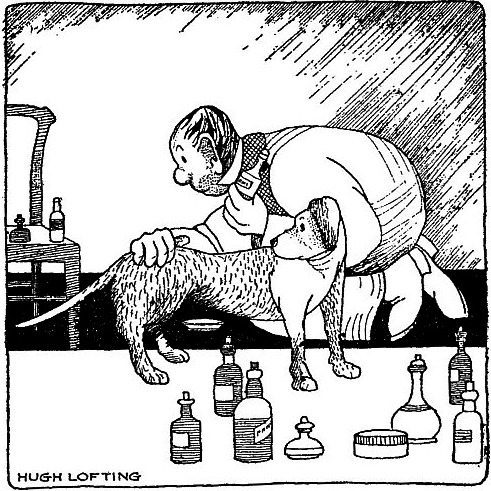

In 2004, NHGRI financed the sequencing of the honey bee, zebra fish, chicken, rat, cow and other organisms. In August 2004, it announced that its Large‐Scale Sequencing Research Network would tackle the genomes of 18 new organisms, including the 13‐lined ground squirrel, the megabat, the microbat, the tree shrew, the bushbaby, the hyrax, the Lorax, the pangolin, the sloth and the Northern white‐cheeked gibbon.^9^

Naomi Bogglehead, Ph.D., A.S.A.P., who agreed to be interviewed under conditions of de‐identification, purchased the food truck from Farfel in 2010 after rotating through several postdoc, teaching assistant, adjunct, affiliate, laboratory assistant and other academic positions at major universities. She explained that when large‐scale sequencing required hundreds of millions of dollars, NHGRI supported it. She did not know where else the money could have come from. But as sequencing costs fell, it became less and less obvious where that kind of money could go.

Bogglehead saw sequencing costs plummet and surmised that her career in genome science was doomed. ‘I invested in a food truck in order to meet my student debt. It was the right decision. Many of my fellow post‐docs worked Illumina HiSeq 2500s and went broke because sequencing had become too cheap to meter’.

‘The less research costs, the less it is worth pursuing’, Farfel explained. ‘What's the “overhead” on a $200 genome?’

Farfel recalled that sequencing costs in 2004 were high enough that NHGRI was able to drop $30 million on Tasha.^10^^,^ 11 Because costs fell, the Broad Institute received only half as much ($15 million) in 2007 to sequence the horse.^12^ Agencourt Bioscience Corp., Beverly, Mass., got only $10 million to sequence the domestic cat. Sequencing costs continued to plummet. It costs about $1000 or less to sequence an animal genome today.^13^

Bogglehead chatted with the NHGRI lunch crowd. She learned that even before the Zucchini centaur, NHGRI faced two new grand challenges. Several of her customers had read an article in *Discover Magazine* (April 2, 2010) that lamented what was called the ‘Yet‐Another‐Genome Syndrome‘^14^ The author wrote, ‘There’s a certain kind of headline I have become sick of: “Scientists Have Sequenced the Genome of Species X!”’. A compelling need to sequence the genomes of many more organisms no longer existed, if it ever had.

The writer grumbled that his e‐mail inbox was deluged daily with press releases of the following form: ‘Scientists have sequenced the genome of species X. Their research, published today in the *Journal of Terribly Important Studies*, will lead to new insights about this important species. Maybe it will even cure cancer or eliminate world hunger!’

Bogglehead was not surprised that genomic data had become more expensive to store than to produce. Researchers estimated that the genomic data with which NHGRI must now cope exceeds the exabytes managed by Facebook, Google, Twitter or YouTube.^15^ There seems to be no legal way to get rid of sequence data, moreover, if they are produced with public funds.

A famous genome scientist is shown on his yacht with sequenced animals watching the Cloud where genomic data are kept.

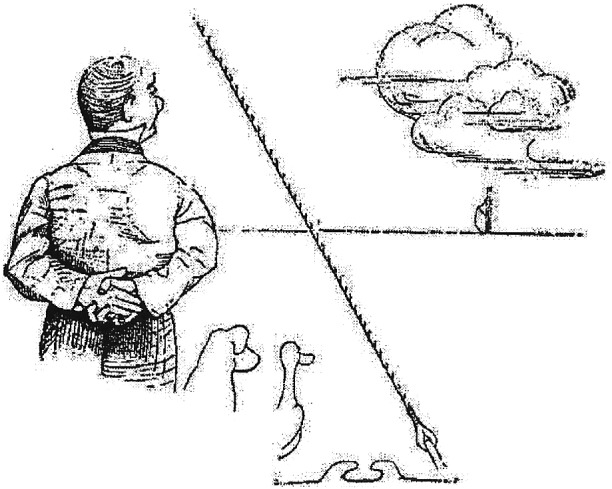

Bogglehead said the lunch crowd described sequence data as ‘exhaust’ since they are generated because they can be and not to test hypotheses. As the exhaust expands, it may spew a million correlations, but no inking of causality. ‘It's not the classic problem of searching for a needle in a haystack by adding more hay. It's that all the needles turn into hay’, Bogglehead said.^16^

Farfel believes there is still a bright future for genome sequencing. ‘The company that sequences your genome for free and links you to all the information available about your variants will create an advertising empire bigger than Facebook or Google’.

The lunch crowd was apprehensive for another reason. They wanted to ‘de‐visit’ the hype that had brought the big bucks to NHGRI. The Large‐Scale Sequencing Research Network was up for another renewal in 2011. The grand challenge was to justify this. In an unsavoury survey of the lunch line, most respondents agreed with the statement, ‘It is economically rational to shutter the Large‐Scale Sequencing establishment as a sunk cost’.

Laboratory technicians who gathered over lunch were concerned not just about the data deluge but also about the lack of progress – what is called ‘translation’ – from byte to bench to bedside. Those who bought bratwurst or salami sandwiches generally thought that medical progress goes the other way – from surprises, successes and failures in a clinical setting to laboratory analyses and then to questions or hypotheses that genomic science might answer.^17^ Science follows medicine. Those who thought the ‘translation’ goes from science to medicine rather than the other way around generally ordered egg salad or corned beef with mayonnaise.

NHGRI's program director for the Large‐Scale program said in a 2010 interview, ‘We have long understood that the ultimate aim is for genomic information to be relevant in the clinic and to individuals’.^18^ Good to know. The Large‐Scale Sequencing Research Network needed about $100 million for each of the next several years to keep on sequencing. The program director said that ‘the main point is that we will continue our successful Large‐Scale Sequencing Program’. Seq and you shall fund.

The 10 000 monkey genome project.

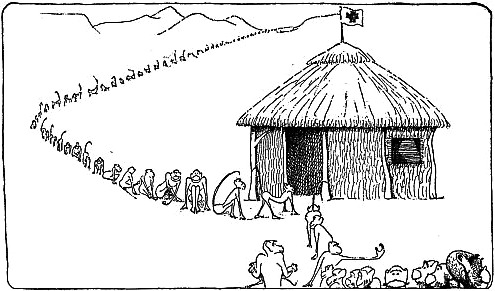

But he also conceded ‘that it is less and less possible to divorce the production of sequence from its analysis and what it is to be used for’. According to many observers, however, this divorce remains altogether possible, even irreconcilable. In a 2018 press release, NIH stated that it had ‘to address a major barrier to incorporating genomic medicine into healthcare, which is a lack of evidence about the relationship between gene variants and diseases’.^19^ Why this lack of evidence? ‘Genomics is a way to do science, not medicine’, said Harold Varmus, then president of the Memorial Sloan‐Kettering Cancer Center in New York.^20^

In 2011, NHGRI announced a new $416 million, four‐year plan, renamed the Genome Sequencing Program, with roughly the same resources as its predecessor, the Large‐Scale Sequencing Program, and roughly the same grantees. ‘With the investments made over the last decade, and the new ones that we are now announcing, a path towards the realization of genomic medicine can be envisioned’, the NHGRI Director said in a statement in December of that year.^21^

A path to the realization of genomic medicine envisioned.

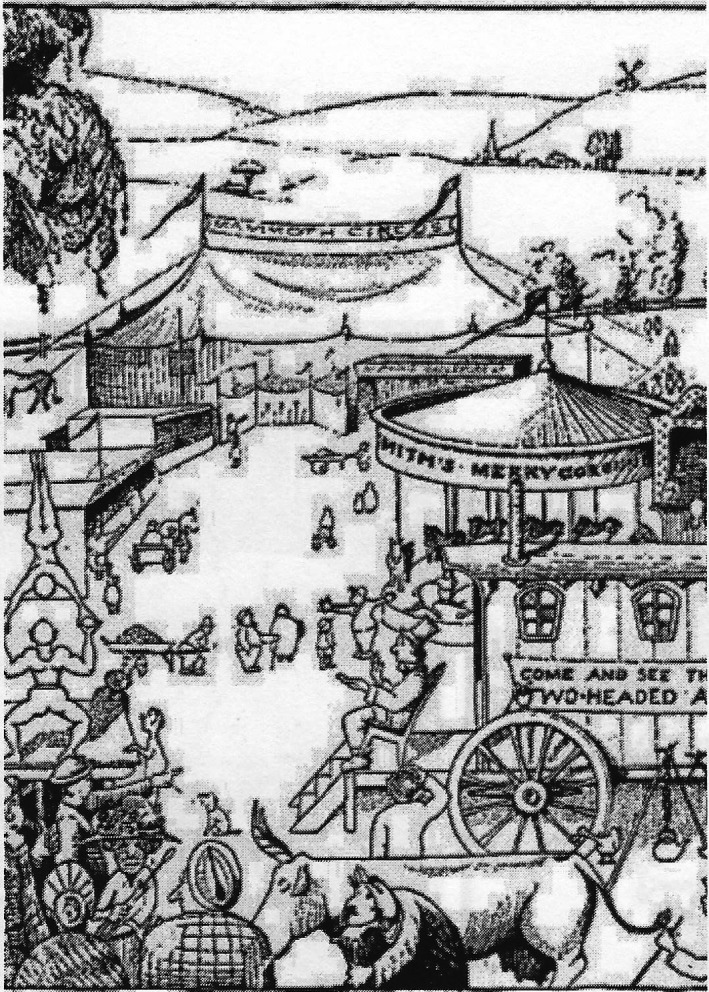

The path towards the realization of genomic medicine had already taken a surprising turn. Garmisch, when he returned from Kuala Lumpur after he was unable to find the conference, received a joyful welcome at the food truck. He had prepared a conference paper on 23andme and other direct‐to‐consumer (DTC) genotyping firms. 23andme genotyped, that is sequenced the genetic variants, of customers who mailed in saliva samples and in return received updates about their levels of risk for 254 heath conditions.^22^ *Time Magazine* in 2008 declared ‘The Retail DNA Test’ to be the ‘Best Invention of the Year’.^23^

‘NHGRI and 23andme compete in the same business’, Garmisch explained, ‘which is to make the connection between genomics and healthcare’. A woman in a laboratory coat, while waiting for a bratwurst, opined, ‘Well, 23andme does not pay “indirect costs” to universities and research centers. It competes on its own dime. This is what made Venter so hated and suspect’.

‘No “indirect costs” – that's unethical!’ laughed a colleague. ‘Next thing you know, they'll produce something that benefits people and makes a profit. Immoral!’

‘The profit motive may do better than the funding motive’, Garmisch said.

While NHGRI re‐upped the Large‐Scale Sequencing Program, the Food and Drug Administration (FDA) warned 23andme that it made unsubstantiated claims because its Personal Genome Service (PGS) linked the genotypes of individuals to health risks and health care. In 2013, FDA ordered 23andme to stop marketing the PGS.^24^

Human genome sequencing, which in 2003 was described as a revolution in medicine and, in 2008, was named the Invention of the Year, in 2013 produced a cease‐and‐desist order. If a path towards the realization of genomic medicine could be envisioned, if evidence about the relationship between gene variants and disease could be found, FDA had not seen any sign of it.

NHGRI confronted another grand challenge – and opportunity – about this time. In 2010, when the folks around the food truck were wondering what NHGRI would sequence next, they chatted about an article recently published in *Genome Research*, ‘Windshield Splatter Analysis with the Galaxy Metagenomic Pipeline’.^25^

In this path‐envisioning research, experimenters on June 23, 2007, at 06:00 h EDT, affixed a 3M 5414 Water Soluble Wave Solder Tape to front bumper of a 2006 Dodge Caravan (‘The Wanderer’) which they drove from State College Pennsylvania to Manchester, Connecticut, where they arrived about 6 h later (Trip A). After they dissolved the tape, they purified, centrifuged and sequenced the biological sample. Later they performed the same experiment on a trip between Portland, Maine, and Moncton, New Brunswick (Trip B).

These researchers identified genome sequences from thousands of plants, insects, and bacteria and calibrated the differences between the windscreen splatter on the two trips.

People waiting for sandwiches quickly saw the funding implications. How did the two splatter communities (Trip A vs. Trip B) differ in their structure and function? If the experiment were repeated with different results, would these represent changes in the Trip A and Trip B splatter communities or entirely different communities? In other words, under what changes in its constituent microbiota does a windscreen splatter community remain the same microbiome and when does it collapse or change into a different one?

‘The genius of terms like “community”, “ecosystem”, “biome” and “network” lies in their inscrutability’, a customer commented. ‘The presumptive need to understand these kinds of concepts, with all their opacity and unintelligibility, will keep us microbiologists in business for a hundred years in defining our concepts – just like ecologists’.

‘If we could base genomic science on concepts this obscure and confused, we'd be looking at all kinds of research opportunities to try to find what they mean’, another enthused.

NIH from the perspective of its fundees

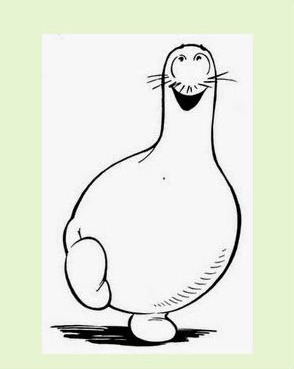

The first replied, ‘This bratwurst is full of bacteria. When they get in my stomach, they become part of me, part of who I am, a second genome. You are what you eat’.

‘That's a billion‐dollar idea!’ exclaimed a third customer. ‘If windscreen splatter forms a functional and identifiable system or community, what about the microbiota in one's nostril or between one's toes? There's no money in calling it “toe jam”. What we call “snot” is actually the human microbial nostril splatter community’.

‘No; I would not say that,’ Farfel offered. ‘I'd call it the Human Microbiome’.

Everyone got the point. The HGP, in the form of the Human Microbiome Project, now had trillions of ‘human’ microbiotas to sequence.

‘I'll bet there are 10 times as many bacterial cells as human cells in the human body. I guess microbes constitute 90 percent of the cells in us. Bacteria “Я” us’, Farfel exclaimed.

‘A 10:1 ratio of microbial to human cells – I like it’, said Garmisch. ‘It's ridiculous and nonsensical, but that's good because it makes the “microbiome” more fundable. It pumps up the numbers; it should become boilerplate in all scientific studies and grant proposals!’

As if on cue, NIH in a press release dated 13 June 2012, stated, ‘The human body contains trillions of microorganisms — outnumbering human cells by 10 to 1’.^26^ It announced $200 million more dollars for the Human Microbiome Project (HMP).

It seemed that every microbiologist looking for HMP support got on the 10:1 bandwagon. Numbers that high worked. A 2015 report released by the National Science and Technology Council (NSTC) noted that annual Federal investment into microbiome research tripled over FY 2012–2014, with more than $922 million invested during this 3‐year period.^27^

An article in the *Boston Globe* reported that during the leadup to the HMP, a microbiologist ‘explained that “we are ten parts microbe, and one‐part human. We are clearly outnumbered”. The figure has been repeated by researchers in scientific journals, books, and TED talks. Scientists joke that it is practically illegal not to cite some version of the figure in a presentation on the human microbiome’.^28^

In 2016, after journalists and others not looking for grants ridiculed boilerplate pronouncements about how ‘we’ are mostly microbial, scientific journals started accepting articles which argued that that the 10:1 ratio was overstated by an order of magnitude.^29^

If we are not outnumbered by the microbial splatter on and in us, what to do with the humongous data exhaust the HMP had created? Different algorithms which pester the data in different ways will suggest thousands of associations and correlations between microbial data and diseases. The folks waiting for sandwiches, however, were not impressed.

Naomi's mother, Lita Bogglehead, age 67, had come in to run her daughter's food truck because Naomi had an interview that day with a commercial genomics company where she hoped to work.

The elder Bogglehead, like a great many seniors, suffered from GERD and acid reflux. ‘A billion federal dollars probably has been spent on studying the gut microbiome community, whatever that is’, the elder Bogglehead observed, ‘but it hasn't done a dime's worth of difference for us seniors. We still belong to the Pepcid Generation’.

‘Every evening after dinner, I sneak up to my room and “drop” antacid’.

*Note: All but one of the illustrations were taken from, The story of Doctor Dolittle, being the history of his peculiar life at home and astonishing adventures in foreign parts, by Hugh Lofting, New York : Frederick A. Stokes Company, 1920. The book can be downloaded at* https://archive.org/details/storyofdoctordol00loft/page/n3. *The shmoo originally by Al Capp is easily identifiable. Any resemblance with Dr. Francis Collins, M.D., Ph.D. is purely coincidental*.

## Conflict of interest

None declared.

## Notes

1 ‘Case Western Reserve Researchers Receive $1.5M NIH Grant to Enhance Guidelines for Ethical Human‐Animal Chimera Research.’ Press Release, Case Western School of Medicine. See http://casemed.case.edu/cwrumed360/news-releases/release.cfm?news_id=1529&news_category=8.

2 ‘An Overview of the Human Genome Project’ December 27, 2012, NHGRI website https://www.genome.gov/12011240/how-nhgri-managed-the-human-genome-project/.

3 Leslie Roberts, ‘Controversial From the Start.’ *Science* 16 Feb 2001: Vol. 291, Issue 5507, pp. 1182‐1188 https://doi.org/10.1126/science.291.5507.1182a. See also Eliot Marshall and Elizabeth Pennisi, “Hubris and the Human Genome,” *Science* 15 May 1998: Vol. 280, Issue 5366, pp. 994‐995. https://doi.org/10.1126/science.280.5366.994.

4 Jerry Menikoff et al., ‘The Common Rule, Updated’, *New England Journal of Medicine* 376(7): 613–615 (2017).

5 For discussion, see Valerie Gutmann Koch, ‘PGTandMe: Social Networking‐Based Testing and the Evolving Research Model’ *Health Matrix* 22: 33–50 (2012).

6 ‘NHGRI Funds Next Generation Of Large‐Scale Sequencing Centers,’ Press Release 2003. https://www.genome.gov/11508922/2003-release-nhgri-funds-next-generation-of-largescale-sequencing-centers/.

7 ‘Dog Genome Assembled.’ NIH press release 2004. https://www.genome.gov/12511476/2004-advisory-dog-genome-assembled/ Sequencing of the dog genome was conducted as part of NHGRI's Large‐Scale Sequencing Research Network, at an approximate cost of $30 million. https://www.genome.gov/17515860/2005-release-researchers-publish-dog-genome-sequence/.

8 Rowan Hooper, ‘Boxer is first dog to have full genome revealed’ *New Scientist* 7 December 2005. https://www.newscientist.com/article/dn8430-boxer-is-first-dog-to-have-full-genome-revealed/.

9 NHGRI Press Release, June 8, 2005, ‘NHGRI selects 13 new targets for large‐scale sequencing program: Strategic mix to include gibbon, bats, sloth and mosquitoes’ https://www.eurekalert.org/pub_releases/2005-06/nhgr-ns1060805.php.

10 NHGRI Press Release ‘Dog Genome Assembled: Canine Genome Now Available To Research Community Worldwide’ July 15, 2004. https://www.sciencedaily.com/releases/2004/07/040715080503.htm; https://journals.plos.org/plosbiology/article/file?id=10.1371/journal.pbio.1002195&type=printable.

11 Sequencing of the dog genome was conducted as part of NHGRI's Large‐Scale Sequencing Research Network, at an approximate cost of $30 million. https://www.genome.gov/17515860/2005-release-researchers-publish-dog-genome-sequence/. See also: https://www.genome.gov/12511476/2004-advisory-dog-genome-assembled.

12 NIH/NHGRI Press Release 2007, ‘Horse Genome Assembled Data on Equine Genome Freely Available to Researchers Worldwide,’ https://www.genome.gov/20519480/2007-release-horse-genome-assembled/. The $15 million effort to sequence the approximately 2.7 billion DNA base pairs in the genome of the horse (*Equus caballus*) was funded by the National Human Genome Research Institute (NHGRI). During 2005, the National Human Genome Research Institute (NHGRI) endorsed a ‘light’ coverage (2×) whole‐genome sequencing strategy for 26 mammals, including *Felis catus*, the domestic cat. https://www.ncbi.nlm.nih.gov/pmc/articles/PMC2045150/.

13
https://www.genome.gov/images/content/costpergenome_2017.jpg.

14 Zimmer, Carl. ‘Yet‐another‐genome syndrome.’ *Discover Magazine, The Loom Science Blog. Also available at:* http://blogs *. discovermagazine. com/loom/2010/04/02/yet‐another‐genome‐syndrome/#. UtZelrQbGg8* (2010). Also: http://blogs.discovermagazine.com/loom/2010/04/02/yet-another-genome-syndrome/#.XBbWMc1OlhE.

15 Hayden, Erika C., ‘Genome Researchers Raise Alarm Over Big Data: Storing and Processing Genome Data Will Exceed the Computing Challenges of Running YouTube and Twitter, Biologists Warn,’ *Nature*, July 7, 2015. http://www.nature.com.mutex.gmu.edu/news/genome-researchers-raise-alarm-over-big-data-1.17912.

16 Wade N., ‘A decade later, genetic map yields few new cures’. *New York Times* June 12, 2010. Available from: http://www.nytimes.com/2010/06/13/health/research/13genome.html?pagewanted=all ‘And many of the genetic variants linked to diseases, some scientists have begun to fear, could be statistical illusions.’

17 Nelson, Richard R., Kristin Buterbaugh, Marcel Perl, and Annetine Gelijns. ‘How medical know‐how progresses.’ *Research policy* 40, no. 10 (2011): 1339‐1344.

18 Matt Jones, ‘NHGRI Plots New Course for Sequencing Centers,’ genomeweb May 26, 2010. https://www.genomeweb.com/sequencing/nhgri-plots-new-course-sequencing-centers?utm_medium=TrendMD&utm_campaign=0&utm_source=TrendMD&trendmd-shared=0#.XBsl7c1OmUm.

19 NIH/NHGRI News Release 2018. ‘NIH‐supported projects remove major obstacles standing in the way of genomic medicine.’ https://www.genome.gov/27572307/2018-news-release-nihsupported-projects-remove-major-obstacles-standing-in-the-way-of-genomic-medicine/ ‘NIH's Clinical Genome Resource (ClinGen) and ClinVar programs address a major barrier to incorporating genomic medicine into healthcare, which is a lack of evidence about the relationship between gene variants and diseases.’

20 Wade N. ‘A decade later, genetic map yields few new cures.’ *New York Times*. 2010 June 12; https://www.nytimes.com/2010/06/13/health/research/13genome.html?pagewanted=all. For further argument to the effect that genomic medicine attempts to salvage genome projects undertaken at enormous cost throughout the world by wresting meaning from them, at which it fails, see Jenny Reardon, *The Postgenomic Condition: Ethics, Justice, and Knowledge after the Genome* (Chicago University Press, 2017) and Isabelle Stenger, *Another Science is Possible: A Manifesto for Slow Science* (Polity Press, 2018).

21 NHGRI Unveils New‐Look Genome Sequencing Program; Plans $416M in Funding. Genomeweb, Dec 06, 2011 https://www.genomeweb.com/sequencing/nhgri-unveils-new-look-genome-sequencing-program?utm_medium=TrendMD&utm_campaign=0&utm_source=TrendMD&trendmd-shared=0#.XB8d981OmUk.

22 Associated Press, ‘FDA Cracking Down on Genetic Tests,’ June 11, 2010. http://www.nbcnews.com/id/37638671/#.XBh5cM1OlhH.

23 Anita Hamilton, *Best Inventions of 2008*, TIME (Oct. 29, 2008), http://content.time.com/time/specials/packages/printout/0,29239,1852747_1854493_18541. 13,00.html [https://perma.cc/TT7L-SE72].

24 U.S. FDA letter 11/22/2013. https://www.fda.gov/ICECI/EnforcementActions/WarningLetters/2013/ucm376296.htm. For an update, see ‘Why You Should Be Careful About 23andMe's Health Test,’ Editorial Board, *New York Times*, Feb 01, 2019. https://www.nytimes.com/interactive/2019/02/01/opinion/23andme-cancer-dna-test-brca.html

25 Pond, Sergei Kosakovsky, Samir Wadhawan, Francesca Chiaromonte, Guruprasad Ananda, Wen‐Yu Chung, James Taylor, Anton Nekrutenko, and Galaxy Team. ‘Windshield splatter analysis with the Galaxy metagenomic pipeline.’ *Genome Research* 19(11) (2009):2144–2153.

26 ‘NIH News Release, June 13, 2012. NIH Human Microbiome Project defines normal bacterial makeup of the body,’ https://www.nih.gov/news-events/news-releases/nih-human-microbiome-project-defines-normal-bacterial-makeup-body . According to NIH, in the ‘normal’ person the bacteria outnumber human cells 10:1. ‘The human body contains trillions of microorganisms — outnumbering human cells by 10 to 1.’

27 ‘FACT SHEET: Announcing the National Microbiome Initiative’ May 13, 2016. https://obamawhitehouse.archives.gov/sites/whitehouse.gov/files/documents/OSTP%20National%20Microbiome%20Initiative%20Fact%20Sheet.pdf.

28 Peter Andrey Smith, ‘Is your body mostly microbes? Actually, we have no idea,’ *Boston Globe*, September 14, 2014 https://www.bostonglobe.com/ideas/2014/09/13/your-body-mostly-microbes-actually-have-idea/qlcoKot4wfUXecjeVaFKFN/story.html?event=event25.

29 Sender, Ron, Shai Fuchs, and Ron Milo. ‘Revised estimates for the number of human and bacteria cells in the body.’ *PLoS Biology* 14, no. 8 (2016): e1002533.

